# Integrated care policy recommendations for complex multisystem long term conditions and long COVID

**DOI:** 10.1038/s41598-024-64060-1

**Published:** 2024-06-13

**Authors:** Christina M. van der Feltz-Cornelis, Jennifer Sweetman, Fidan Turk, Gail Allsopp, Mark Gabbay, Kamlesh Khunti, Nefyn Williams, Hugh Montgomery, Melissa Heightman, Gregory Y. H. Lip, Michael G. Crooks, W. David Strain, Antony Loveless, Lyth Hishmeh, Natalie Smith, Amitava Banerjee

**Affiliations:** 1 Department of Health Sciences, University of York, York, UK; 2 Hull York Medical School, (HYMS), University of York, York, UK; 3 Institute of Health Informatics, University College London, London, UK; 4 Royal College of General Practitioners, London, UK; 5 Department of Primary Care and Mental Health, University of Liverpool, Liverpool, UK; 6 Diabetes Research Centre, University of Leicester, Leicester, UK; 7 Department of Medicine, University College London, London, UK; 8 University College London Hospitals NHS Foundation Trust, London, UK; 9 Liverpool Centre for Cardiovascular Science at University of Liverpool, Liverpool John Moores University and Liverpool Heart and Chest Hospital, Liverpool, UK; 10 Danish Center for Health Services Research, Department of Clinical Medicine, Aalborg University, Aalborg, Denmark; 11 Hull York Medical School, University of Hull, Hull, UK; 12 Hull University Teaching Hospitals NHS Trust, Hull, UK; 13 Diabetes and Vascular Medicine Research Centre, Institute of Clinical and Biomedical Science and College of Medicine and Health, University of Exeter, Exeter, UK; 14 PPI Member for STIMULATE-ICP Consortium, London, UK; 15 Department of Cardiology, University College London Hospitals NHS Trust, London, UK; 16 Department of Cardiology, Barts Health NHS Trust, London, UK

**Keywords:** Health policy, Health services

## Abstract

The importance of integrated care for complex, multiple long term conditions was acknowledged before the COVID pandemic but remained a challenge. The pandemic and consequent development of Long COVID required rapid adaptation of health services to address the population’s needs, requiring service redesigns including integrated care. This Delphi consensus study was conducted in the UK and found similar integrated care priorities for Long COVID and complex, multiple long term conditions, provided by 480 patients and health care providers, with an 80% consensus rate. The resultant recommendations were based on more than 1400 responses from survey participants and were supported by patients, health care professionals, and by patient charities. Participants identified the need to allocate resources to: support integrated care, provide access to care and treatments that work, provide diagnostic procedures that support the personalization of treatment in an integrated care environment, and enable structural consultation between primary and specialist care settings including physical and mental health care. Based on the findings we propose a model for delivering integrated care by a multidisciplinary team to people with complex multisystem conditions. These recommendations can inform improvements to integrated care for complex, multiple long term conditions and Long COVID at international level.

## Introduction

Efforts to establish integrated care for people with complex, multiple long term conditions (also termed multimorbidity) have been made since the 1990s because of the rise of long term conditions in all age groups and the associated costs^1,2^. Integrated care pathways (ICPs) provide care for people who need input from a combination of providers, such as primary care and specialist general hospital care for long term conditions^3,4^. The NHS proposed to provide integrated care using structured, multidisciplinary care plans coordinated across specialties, investigations, treatments and rehabilitation to improve outcomes for patients with long term conditions and complex needs^5^. This was achieved through disease management and chronic care models such as collaborative care for single disease models or care pathways for conditions such as diabetes, cardiovascular disease, chronic obstructive pulmonary disease (COPD) or rheumatoid conditions^6–11^.

More recent efforts aimed to incorporate integrated approaches into the care of complex, multiple long term conditions. These are chronic conditions affecting multiple systems and have a significant impact on life, requiring stepwise approaches to interventions and subsequent monitoring. Those treatments encompass a range of interventions such as medications, surgical interventions, psychotherapy, education about the condition and self-management as well as lifestyle interventions, delivered by a multidisciplinary team^12,13^. Initially they were especially developed for older people^14^, but evolved towards developing integrative approaches to care management including for comorbid somatic and mental disorders^15–24^. The COVID-19 pandemic resulted in a surge in new long term conditions which placed additional strain on health services worldwide, combined with service provision for pre-existing conditions having to be maintained^2^. This has reinforced the ongoing and urgent need for integrated care.

Long COVID is a new long term condition, which has been defined by the World Health Organization as ‘*the continuation or development of new symptoms *3* months after the initial SARS-CoV-2 infection, with these symptoms lasting for at least *2* months with no other explanation’*^25^. Long COVID cannot, at present, be cured, but is controlled by medication or other treatments^26,27^. It affected 2 million people in the UK in 2023^28^ and at least 65 million globally^29^. It is a complex, heterogeneous, multisystem condition which significantly impacts the lives of those affected. Research indicates that 57% of people with Long COVID experience at least one persistent symptom 12 months post-infection^30^. Since 2020, the NHS has set up more than 100 Long COVID services across England^31,32^. Combined with the recent establishment of integrated care boards aiming to improve the quality of care and support for those with complex multiple long term conditions^3^, the development of Long COVID services provides a timely opportunity to inform the optimal set-up of ICPs for complex multiple long term conditions, incorporating learning from the establishment of Long COVID care pathways. An example of potential models for ICPs managing recovery in Long COVID and other LTCs is shown in Table 1 below^33^.

**Table 1:** Potential models for ICPs managing recovery in Long COVID and other LTCs (copied with permission from^33^).

Model	Example condition(s)	Recovery time	Managed by	Approach
Model 1	Community acquired pneumonia (CAP).	This may take 6 months to fully recover in terms of fatigue (NICE guideline.)	Primary care teams and community General Practitioners (GPs). There is a NHS CAP CQUIN aiming to support discharge from the hospital and safe follow up of these patients. Follow up imaging is usually arranged by secondary care.	Currently there is no well-developed integrated care pathway but there could be a chance to identify how to identify CAP follow up better based upon Long COVID care experiences. They support the patient through their recovery with the length of complete recovery and the ramifications for work often underestimated.
Model 2	Post myocardial infarction, significant musculoskeletal injury.	Taking a medium course to resolution, e.g., 1–2 years.	Multi-disciplinary team (MDT) driven and mostly provided in rehabilitation clinics.	Rehabilitation approach, personalised to the individual including a biopsychosocial approach to care, with physiotherapy and medical attention to address anxiety and depressive symptoms.
Model 3	A chronic disease like type 2 diabetes or stroke.	It is managed but often recovery is not complete.	Usually managed in primary or community care, by GPs and diabetes nurses, or in the hospital setting.	Escalation of a small proportion with complex needs being managed in a specialist setting.
Model 4	COPD, rheumatoid arthritis.	A chronic condition that may have high disability with tendency for relapses/ exacerbations.	Limited care provision, mostly based in primary care with exacerbations increasingly managed in hospital in later stages. Growing emphasis on need to improve community diagnostics and where pulmonary rehabilitation is a key evidence-based treatment.	COPD is a condition that shares breathlessness as an important symptom with Long COVID, where pulmonary rehabilitation is a key evidence based treatment and supporting self-management is a key goal. Impact on function, breathlessness and psychological wellbeing as in Long COVID. Both conditions have a relapsing course of symptoms that may benefit from prompt intervention. There is growing emphasis on the need to improve community diagnostics.
Model 5	Comorbid mental disorders and other LTC; for example, COPD in patients with mental disorders, often related to smoking, or depression in diabetes patients.	These are in general chronic conditions with high disability. There is an unmet clinical need here.	Mental disorders have case management, crisis teams, psychiatry follow up, but they do not identify physical health needs of their patients, such as respiratory issues. And clinics for somatic conditions can have short-term treatments available for psychological treatments but there is a lack of available long term integrated treatment.	There are pilot playgrounds for dedicated respiratory clinics for patients with mental illness across the country. Similar pilots exist for diabetes and depression—either community-based or hospital-based.
Model 6	Encompassing multi-morbidity (i.e. more than two LTCs) as well as a spectrum of symptoms that do not fit into a usual pattern for diagnosis of a single disease i.e. Medically Not Yet Explained Symptoms (MNYES) or both, crossing the mental health and physical health divide.	The perceived burden of disease is high. These are conditions requiring a multisystem approach.	No current consistent pathway of care exists. Consultation, collaborative care and decision aids supporting health care providers to provide ICP would be possibilities to link primary, community and specialist health care settings.	These patients are highly likely to benefit from an ICP. This would be best served with a flag up system approach which is for people who don’t quite meet full diagnostic criteria in one condition but almost meet it in many conditions. This would be labelled as MNYES but disease burden is high and there is a need to integrate physical and psychological health care provision.

The approach that was taken in the Long COVID clinics enforced many aspects of integrated care pathways that had been theoretically defined but rarely implemented before the pandemic. The sense of urgency and the need to work swiftly on this obviously multisystem condition forced the swift development of integrated care models in Long COVID clinics. The learning from this experience could provide insights for the development of integrated care pathways for other long term conditions, that had until then remained underdeveloped.

To-date, research has not considered learning from Long COVID health care systems development, to inform integrated care for complex, multiple long term conditions. Additionally, as the set-up of Long COVID clinics could differ by region, it would both be relevant to understand how to better manage Long COVID, and how to better use ICPs for all diseases.

Both the literature on Long COVID and on the importance of integrated care for long term conditions are limited. Moreover, how such care should be implemented depends on policies in the respective countries, that may differ. Therefore, a Delphi study to be conducted amongst experts in the domain of long term conditions, Long COVID and integrated care seemed the right way to proceed to develop priorities for integrated care pathways for Long COVID and long term conditions. Relevant experts were considered to be healthcare providers as well as care users with experience in the conditions of interest. A Delphi study uses a structured method for collecting the opinions of experts (called panelists), concerning a subject of their expertise. This is done anonymously, with each voice counting equally and not being subject to social pressure to adhere to a dominant vision. The essence is an exploration of expert views on a particular topic and then giving the option to the experts to react to the input of the other experts in an iterative procedure which can comprise several rounds of enquiry. Following an initial round of information gathering, a summary of expert opinions is provided to panelists to inform the next round, and so on until consensus is achieved^34,35^.

This article reports the findings of a Delphi consensus study which was conducted in line with the NHS England commitment to improve the involvement of patients in healthcare^36^. The Delphi research group believe that the patient viewpoint can enhance the quality of research and care provision; as such we chose a definition of integrated care that is close to the experience of patients with sometimes multiple long term conditions: “I can plan my care with people who work together to understand me and my carer(s), allow me control, and bring together services to achieve the outcomes important to me”^37^.

The aim of our study is to inform the optimal set-up of ICPs for complex multiple long term conditions, incorporating learning from the establishment of Long COVID care pathways, and how to better manage Long COVID. To this goal, we report the findings of a Delphi consensus study based on patient and health care provider experience.

We explored:

1. Key enabling elements for effective ICPs for Long COVID and strengths of existing ICPs for long term conditions.
2. Which part of an ICP model for Long COVID might be transferred to long term conditions without sufficiently developed care pathways, and how could they be integrated.

## Results

In an 18-month process, following three conferences of the expert group and two surveys providing 461 patient and 180 health care professional experiences with long term conditions (n = 365) or Long COVID (n = 276), this study yielded priorities for integrated care for Long COVID and for long term conditions. Most patients reporting on long term conditions suffered from multimorbidity or complex multiple long term conditions.

### Round one

Table 2 provides demographic characteristics for participants of Surveys 1 and 2. As round 1 was intended to gather information about important characteristics of integrated care pathways (ICPs) for Long COVID and for long term conditions, a detailed description of the responses from survey 1 and how they informed survey 2 questions is available via this link https://www.york.ac.uk/media/healthsciences/documents/research/mentalhealthresearch/STIMULATE-ICP%20Delphi%20Responses%20Table.pdf.

**Table 2:** Table of survey participant demographics.

	Survey 1	Survey 2
	N = 283	N = 197
Gender (%)		
Male	45 (16)	75 (38)
Female	127 (45)	119 (60)
Non-binary/third gender/other	2 (< 1)	2 (1)
Prefer not to say	5 (2)	1 (< 1)
Missing	104 (37)	0
Age		
Mean (sd)	44.80 (11.31)	41.25 (12.43)
Range	21–75	18–74
Experience (%)^a^		
LC patients	222 (78)	68 (35)
LTC^a^ patients	123 (43)	48 (24)
HCP experience	95 (34)	85 (43)
Carer	0	7 (4)
No experience LC or LTC	0	41 (21)
Location (%)		
England all areas	111 (39)	180 (91)
N. Ireland	3 (1)	1 (< 1)
Scotland	14 (5)	11 (6)
Wales	3 (1)	5 (3)
Missing	111 (39)	0
Ethnicity (%)		
Asian/Asian British	4 (1)	33 (17)
Black, Black British, Caribbean	1 (< 1)	15 (8)
or African		
Mixed or multiple ethnic groups	5 (2)	13 (7)
Other ethnic group	5 (2)	5 (3)
White	162 (57)	131 (67)
Missing	106 (37)	0
Work status (%)		
Full-time	86 (30)	103 (52)
Part-time	37 (13)	44 (22)
Self-employed/freelance	11 (4)	9 (5)
Student	3 (1)	8 (4)
Retired	5 (2)	8 (4)
Unemployed	9 (3)	9 (5)
Volunteer	1 (< 1)	1 (< 1)
Other	22 (8)	10 (5)
Carer	0	1 (< 1)
Prefer not to say	5 (2)	2 (1)
Missing	104 (37)	2 (1)

*LC* Long COVID, *LTC* long term condition, *N* the actual number of participants that completed the surveys.

^a^Survey 1: 178 (63%) people had experiences relevant to a single group, 105 participants (37% total sample) had mixed experiences. Survey 2: 125 (63%) people had experiences relevant to a single group, 72 participants (37% total sample) had mixed experiences.

The professional profile of the moderator and expert panel is described in Delphi participants.

### Round 2

In round 2 (Establishing consensus and ranking), Survey 2 findings identified ten statements which achieved 80% consensus; these were the same for both Long COVID and long term conditions and are presented in Table 3. Color coding illustrates the similarities and differences in rank order between statements for Long COVID care and long term condition care.

**Table 3:**
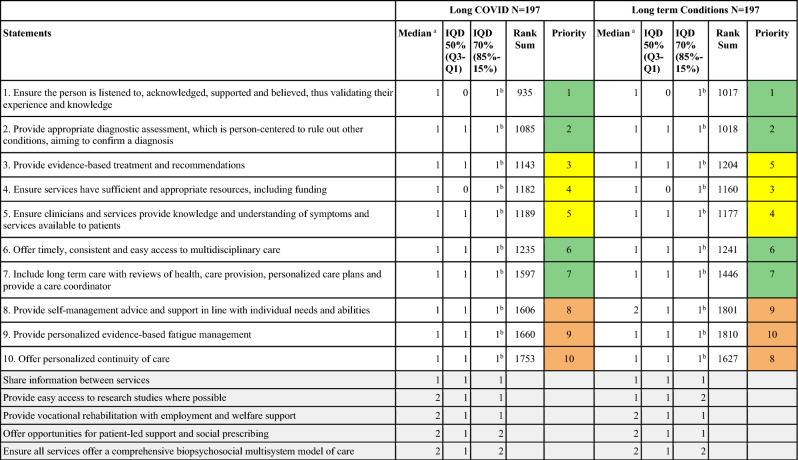
Delphi consensus and prioritization results.

^a^1 = Strongly agree, 2 = Agree, 3 = Do not agree or disagree, 4 = Disagree, 5 = Strongly disagree.

^b^Items which retain consensus at 80%

*LC* Long COVID, *LTC* long term condition.

Color key: Green = identical priority ranking for LC and LTC; Yellow = similar orderin﻿g for statements ranked 3–5 for LC and LTC; Orange = similar ordering for statements ranked 8–10 for LC and LTC.

The top two statements for both Long COVID and long term conditions were:

1. Ensure the person is listened to, acknowledged, supported and believed, thus validating their experience and knowledge.
2. Provide appropriate diagnostic assessment, which is person-centered, to rule out other conditions, aiming to confirm a diagnosis.

The following three statements were ranked between 3 and 5 for Long COVID and other long term conditions:

3. Provide evidence-based treatment and recommendations
4. Ensure services have sufficient and appropriate resources, including funding
5. Ensure clinicians and services provide knowledge and understanding of symptoms and services available to patients

Numbers 6 and 7 are ranked the same for both conditions, and the last three are ranked between 8 and 10.

### Round 3

Subsequently in round 3 (Developing policy recommendations), fifteen policy recommendations were developed based on the expert panel discussions about those ten statements. These recommendations concern four domains: diagnosis, resources, training and research. Recommendations, outlined in Table 4, include sufficient time with clinicians to assess the problem or condition and offer timely access to diagnostic investigations; research to provide evidence-based treatment and rehabilitation; appropriately resourced services with funded time for specialist consultant contributions to multidisciplinary care and routine follow-up appointments; and training to increase the knowledge and understanding of health care professionals, communities and employers.

**Table 4:** Description of policy recommendations for integrated care which were developed from the Delphi statements.

Diagnosis (priority 2)	Resources (priorities 4,6,7,9,10)	Training (priorities 1,5,8)	Research (priority 3,8)
Microlevel			
There is a need to develop screening and assessment protocols for Long COVID and long term conditions.	Appropriate, routine follow-up appointments should be offered to patients by a HCP with specialist knowledge about their condition.	Continuing Medical Education for qualified medical and allied health professionals should highlight how integrated care models can support people with Long COVID and other complex, multisystem, long term conditions.	There is a need to explore evidence-based treatments and rehabilitation interventions (such as fatigue management) for Long COVID and other complex MLTCs.
Mesolevel			
Structural availability of consultation with specialists to advise primary care multidisciplinary teams about diagnosis and treatment.	Shared electronic health records, providing opportunities for discussion of care and for joint consultation, are needed.	In the curricula of medical and allied health professional students attention should be paid to how integrated care can support people with Long COVID and complex MLTCs.	Research into the cost-effectiveness of service redesigns to provide integrated care is needed.
Macrolevel			
Diagnostic centers to improve access to diagnostic investigations relevant for Long COVID and other complex MLTCs.	Sufficient funding should be made available to local services for the completion of comprehensive assessments; resourced with workforce availability to reduce the risk of opportunity costs to the detriment of other groups and care needs.	Public health campaigns to raise awareness about Long COVID and MLTCs, including the symptoms and experiences of those affected should be prioritized. Such campaigns should clarify information around self-management, when to seek formal health care support, and how communities and workplaces can support those experiencing symptoms.	Research funding: Integrated care models for Long COVID and complex MLTCs should be prioritized in research funding decisions. Trials evaluating new treatments for Long COVID are needed as well.
Diagnostic centers should have low thresholds to access relevant diagnostic tests and timely specialist reports of findings and significance.	There is a need for a society or national organization that develops ways for existing societies and organizations to work together on MLTCs and Long COVID.	There should be a new guideline on managing complex MLTCs and complexity/ multimorbidity.	

*MLTC* Multiple Long Term Conditions. Numbers of relevant priorities are listed in the Table column captions.

## Discussion

Key priorities identified in this work center around evidence-based support, provided by services with appropriate resources (including funding) and clinicians who are knowledgeable and understanding of symptoms and aware of the available services. Care should be offered in in a timely and consistent manner, with easy access through multi-disciplinary teams. Healthcare should include long term support, reviews, personalized care plans and a care coordinator. Self-management advice should be provided in line with individual needs, as should personalized evidence-based fatigue management. Personalized continuity of care was also prioritized. The findings of this Delphi study show that priorities identified for long term conditions are very similar to those for Long COVID, a recently established condition. Both patients and health care providers envisioned priorities with a high consensus rate of 80%. The two top priorities were *to ensure the person is listened to, acknowledged, supported and believed, thus validating their experience and knowledge;* and *to provide appropriate diagnostic assessment, which is person centered, to rule out other conditions, aiming to confirm a diagnosis.*

These findings indicate the importance of validating the patient experience and providing adequate diagnostics when planning care for complex conditions*.* The Health Committee of the House of Commons stated that in view of multimorbidity, the person should be treated, not the condition, and that the single-disease framework on which the NHS mainly operates is less successful in treating people with complex multiple long term conditions^38^. The patient and healthcare provider priorities which we identified led to the development of policy recommendations which resonate with this statement.

It is not surprising that priorities concerned with taking the patient seriously and performing adequate diagnostic assessments were important for Long COVID, as this is a new condition with many diagnostic uncertainties, and patients were actively discouraged from seeking help from their primary care physician in the initial phase of the pandemic to reduce cross-infection risks^39–41^. In contrast, the fact that patients and clinicians gave the same priority and ranking for other long term conditions was an unexpected and novel finding. The COVID-19 pandemic resulted in organizations struggling to meet previous objectives for long term condition management^42^. Therefore, these priorities could reflect the strain on current health care services. On the other hand, the wish to prioritize the validation of patient experiences and the provision of adequate diagnostics could indicate that the challenges of implementing integrated care into routine practice pre-dated the COVID-19 pandemic^43^. Given the level of importance placed on diagnosis by Delphi participants in this study, Box 1 shows an example of how a single response made by a participant with lived experience of Long COVID was categorized at each stage of this Delphi, and subsequently contributed to policy recommendations relating to Diagnosis.

**Box 1:** Example of the transition from one original participant statement to final policy recommendations.

Original Survey 1 response: *‘An in-depth conversation should be the first step to finding out what kind of care is necessary. Questions about symptoms alongside those about how symptoms are affecting life and relationships. From there, the patient can be directed to the right support. Ideally this support can be provided in the same place and would run concurrently’.* (Patient with Long COVID experience)
Phase 1 i) Initially organized by the research team under: *Provide diagnostic assessment.* ii) Then organized by small groups of expert panel members, led by a moderator panel member under: *Provide appropriate diagnostic assessment which is person centred to rule out other conditions.* iii) This original response ultimately contributed to statement: *Provide appropriate diagnostic assessment which is person centred to rule out other conditions.* Moderator panel members approved this statement to go into survey 2.
Phase 2 Statement achieved consensus and was ranked as Priority 2 during Survey 2.
Phase 3 Following extensive discussions with moderator panel and expert panel groups, the original responses which informed the statement ‘Provide appropriate diagnostic assessment which is person centred to rule out other conditions’ contribute to the final policy recommendations relating to Diagnosis: Microlevel: There is a need to develop screening and assessment protocols for Long COVID and long term conditions. Mesolevel: Structural availability of consultation with specialists to advise primary care multidisciplinary teams about diagnosis and treatment. Macrolevel: Diagnostic centers to improve access to diagnostic investigations relevant for Long COVID and other complex MLTCs. Diagnostic centers should have low thresholds to access relevant diagnostic tests and timely specialist reports of findings and significance.

The expert panel from this study recommended that the experiences with the newly formed Long COVID clinics could offer an opportunity to innovate integrated care within a complex care pathway. Long COVID clinics bring multiple providers together to meet needs broader than has historically been possible for many complex long term conditions, and evaluations of Long COVID clinics indicate that accessing a virtual multidisciplinary team without additional referrals has enhanced the ‘one team’ approach^44^. This has facilitated knowledge exchange and enhanced the integration of primary and specialist care for Long COVID; it also minimized referrals to single-specialty services. In addition, this approach is able to facilitate appropriate allocation of resources for further investigation, action plans for correct diagnosis provision and treatment for personalized complex care management^45^. The policy recommendations outlined in Table 4 reflect the agreed statements and concern four aspects of care: diagnostics, resources, training of health care providers, and research to develop evidence-based treatment and rehabilitation programs for Long COVID, and also to evaluate integrated care models and their cost-effectiveness for complex long term conditions. Consequently, in this paper we propose an overall model of care which could be replicated and further developed to ensure consistency in the delivery of services nationally, and to inform similar changes internationally.

This study suggests that more effective coordination and workforce planning, to support specialist input into care plans, should be prioritised in integrated care. There is a need to provide resources for the introduction of multi-disciplinary team case discussions and structurally embedding consultation with specialists into primary care, including for mental health. Introducing this for complex, multiple long term conditions would be novel and would reduce multiple onward referrals. Other innovative recommendations would be: (i) enhanced workforce training to support skills transference and interdisciplinary learning; (ii) support by local public health teams for public health campaigns; (iii) tracking access to services and patient outcomes of service provision; and (iv) benchmarking of quality of care and service improvement. Inclusion of public health contributions and workforce training is not typically incorporated into integrated care pathways; however, these align with the broader objectives of disease management programmes to improve health or prevent disease, and to enhance the cost effectiveness of an ICP.

Integrated care has previously been located in primary care or the hospital setting^46,47^. New post-pandemic aspects of integrated care, indicated by the expert panel and based on the findings of this work, include resource provision for better integration of specialist care relevant to multiple conditions within primary care, such as the structural embedding of secondary care consultation specialists without the need for separate referrals. Additionally, the innovation of virtual multidisciplinary teams supported by primary, secondary, tertiary, community, physical and mental health care specialists and their subsequent implementation offers an opportunity to broaden the number of integrated care pathways beyond Long COVID to other complex conditions and to provide care to community-managed patients without requiring multiple referrals^48,49^. The leading principle should be that access to care is not enough; people need access to effective care and treatments which could be attained by personalization of care within a model of vertical integration between primary and specialist care, the support of multidisciplinary teams and monitoring by a care manager with expertise relevant to the care of complex multiple long term conditions.

The health economic impacts of implementing our proposed solutions require further evaluations to determine the value of having the necessary expertise available in meetings, compared with the cost of consultant time, multiple referrals and discharges, opportunity costs for staff and associated societal costs for fragmented care like loss of productivity and travel. To prioritize a person’s comprehensive needs in a single ICP requires broadening the available options and incorporating integrated psychiatric treatment and vocational rehabilitation. This must balance with competing costs and workforce demands. The current crisis in health care and the increasing burden of complex, multiple long term conditions demands urgent solutions, which may mean increasing resources. However, given the limited resources and the challenges to dealing with complex, multiple long term conditions that have become apparent, it should maximize the use of current resources towards integrating services, avoiding stacking treatment for each condition by making lean, personalized treatment programs. In addition, diagnostic centers available for some health conditions could be expanded to cater for a wider variety of complex conditions; better links between these centers and the broader health care environment could be made.

This work was completed in the UK; but, given the pandemic context and international efforts to establish integrated care, we deem the results to be relevant internationally. Our findings align with international recommendations for multiple long term condition care since the start of the COVID-19 pandemic. In Australia, increased primary care funding has been identified to increase the length of consultations to enable Primary Care Physicians to fully understand the presenting problem(s)^50^. In the USA, 40 Long COVID clinics were established and calls for changes to service configuration have been made to support the care of people with complex multiple long term conditions, integrating appropriate services to provide coordinated patient-centered care^23,51^. In Europe, studies have reported some of the logistical adaptations needed to provide these services in the context of the pandemic, both for comorbid mental disorders in the general hospital setting^52^ and for the diagnosis of Long COVID in the primary care setting^53,54^. In Africa, health care organization, delivery system design, a clinical information system, self-management support, community linkages, and trained educators and decision support to link informed, activated patients and prepared proactive teams are identified as critical components of collaborative care for complex multiple long term conditions^55^. However, it should be noted that these studies were performed from the researchers’ and providers’ perspective and did not include patients’ views. Our study now presents the view of patients and health care providers working across primary, secondary and acute specialty care including specialty mental health care on priorities for integrated care.

This study is of great clinical need; Long COVID is a major problem for health care systems and societies following the COVID-19 pandemic. Representation of patient and health care providers’ views is a novelty, and the study’s large sample size and high consensus rates are strengths. The Delphi consensus method we adopted incorporated data from a diverse sample of patients and clinicians with a wide range of Long COVID and long term condition experiences from all devolved nations of the UK. It combined more than 1400 responses from nearly 500 survey participants and was supported by patients, clinicians, and charitable and professional organisations across the UK. This enabled the integration of views from different stakeholder groups, going beyond similar research where patients’ involvement was not incorporated at all or any stages^52^. Incorporating views relevant to Long COVID and complex multiple long term conditions and the findings highlighting the similarities in health care priorities is novel and another strength of the study. The 80% level of consensus, which identified the same top 10 statements as priorities for long term condition care as for Long COVID care, is very high when compared to consensus levels in most Delphi studies indicating substantial agreement that the prioritised statements are essential to address during ICP development which is another strength.

Limitations are that despite efforts to recruit a diverse sample, the vast majority of respondents to survey 1 were female and of white ethnicity, similar to other UK health-related research^56^ and to the demographics of people attending Long COVID clinics. It is thus possible that priorities pertinent to individuals from alternative backgrounds may not have been reflected in the statements presented. For survey 2, we took more measures to guarantee a diverse sample regarding ethnicity and gender by additional purposive sampling^57^. Nevertheless, our sample was only drawn from the UK and mainly, but not exclusively, recruited the public respondents via online systems, which may introduce bias relating to the digital divide and health pressure groups. In contrast, digital access for health care providers should be less of a distorting factor. Despite these limitations regarding the generalizability of the findings, we believe the findings can be relevant to health care systems internationally.

Given the paucity of integrated care guidelines for complex, multiple long term conditions, this study reinforces the need to continue striving to provide care which is patient-centred, ongoing and supported by evidence. This paper starts to meet this need by offering concrete recommendations to support the implementation of interdisciplinary care for complex long term conditions and Long COVID in routine practice; for example, by the inclusion of multidisciplinary teams to provide interventions, and by coordinated, integrated plans for treatment and long term support.

In the context of integrated care for Long COVID and complex, multiple long term conditions, people need access to care and treatments that work, not just more access. They need diagnostic procedures that support the personalization of treatment in an integrated care environment, avoiding stacking treatment for each condition by making lean, personalized treatment programs supported by a care manager, a virtual multidisciplinary team and specialist consultants as needed. This model for delivering integrated care can be attained by vertical integration between primary and relevant specialist care, delivered by a multidisciplinary team including physical and mental health care, that may convene virtually. Actioning the policy recommendations from this research may help ensure appropriate care provision for the growing number of people with complex, multisystem long term conditions requiring management. With a gap in health care provision for Long COVID in many countries, and the similar problems identified by this study for complex multiple long term conditions, our recommendations offer guidance for international communities to develop similar integrated care approaches appropriate both for Long COVID and for refining health care provision for complex multiple long term conditions.

## Methods

### Design

Since the invention of the Delphi method in the 1950s^58^ a commonly used variation of the Delphi method is the estimate-talk-estimate Delphi method that combines assembling of expert opinions on an anonymous basis during surveys with open exchange during workshops by a facilitator or moderator panel^59^; this approach was adopted for the current project. This Delphi consensus study involved two surveys: an exploratory survey followed by a consensus and ranking survey, alternating with expert panel meetings from February 1 2022 to March 30 2023. It sought to identify integrated care priorities and to inform policy recommendations. We report the findings following the Guidance on Conducting and REporting DElphi Studies (CREDES)^34^ and a checklist outlining where they can be found (Supplementary Information).

### Scope

The plan and scope of the work was discussed and agreed with expert panel members at the first meeting. Adults (aged ≥ 18 years) residing in the UK were eligible to participate. Following input from the STIMULATE-ICP Patient and Public Involvement (PPI) group^33^, regarding the definition of complex multiple long term conditions for consideration in the IC priorities, input in the survey on fatigue, as a symptom, was in scope. However, given an ongoing discussion between patient groups on whether Long COVID would or would not differ from myalgic encephalomyelitis or chronic fatigue syndrome (ME/CFS), and the wish of the PPI group to focus on Long COVID, ME/CFS was considered out of scope.

### Delphi team

This work involved a research team, a moderator panel and an expert panel.

- The research team (n = 4) consisted of academic researchers (JS, FT, CFC) and a coordinator (NS). This group worked across all aspects of the project.
- The moderator panel included two General Practitioners and two PPI members (MG, GA, EA, LH) drawn from the STIMULATE-ICP consortium. This group met with the research team on 20 occasions (typically fortnightly) throughout the project to oversee all aspects of the research.
- Expert panel members were selected by the moderator panel and research team. The aim for this group was to include people with clinical expertise as Health Care Providers (HCP) or lived experience as patients, of Long COVID, other LTCs such as cardiovascular disorders, type 2 diabetes, mental disorders and MNYES, or multi-morbidity; 5 of each group with a minimum of 25.

A snowballing method and a maximum variation approach to selection were followed to recruit expert panel members. Patient expert panel members were recruited from peer support groups, previous research project PPI groups, charities relating to health conditions (including Diabetes UK, Guts UK, British Pain Society, Epilepsy Action, British Thyroid Foundation, Graham Hughes International Charity-antiphospholipid syndrome) and NHS trust patient involvement networks. Based on self-reported diagnosis, fifteen people with lived experience of Long COVID or long term conditions such as cardiovascular disorders, type 2 diabetes, mental disorders and medically not yet explained symptoms (MNYES), or multi-morbidity were included (total n = 15: Long COVID experience = 9, long term condition experience = 10). Six patient expert panel members had only Long COVID experience, 12 had only (diverse) long term condition experiences and 11 had both Long COVID and long term conditions. Patient expert panel members included people who lived with single and multiple long term conditions. Overall, the patient expert panel members had experience of the following long term conditions: fibromyalgia, POTS, diabetes, endometriosis, psoriatic arthritis, cluster headaches, asthma, COPD and mental disorders including anxiety, depression, bipolar disorder, Functional Neurological Disorder and PTSD.

HCP expert panel members were medics recruited from professional clinical networks, medical trusts and the Royal College of General Practitioners, as well as contacts from the STIMULATE-ICP consortium (total n = 14: working with Long COVID = 8, working with long term condition = 14). Specialties included Primary Care (n = 4), Psychiatry (n = 3), Cardiology (n = 2), Diabetes (n = 1), Geriatrics (n = 1), Rehabilitation medicine (n = 1), Stroke (n = 1) and COPD (n = 1).

### Data collection and analysis

#### Round 1: gathering information

Survey 1 collected anonymous electronic data using the Qualtrics survey platform^60^. Based on input from the expert panel and the moderator panel, the survey included questions about (1) demographic factors (age, gender, ethnicity), (2) relevant disease experience as patient or clinician; (3) experiences of initiating care, referrals, treatment(s) offered and received; (4) challenges and advances for clinical care, (5) knowledge gaps and policies, (6) possible improvements to services, and (7) the transferability of care models to other conditions. These topics were explored for Long COVID and for long term condition integrated care pathways (ICPs). Questions were presented to participants in blocks, depending on their self-reported experience as a person with lived or professional experience of Long COVID or long term conditions. Participants were able to respond to multiple question blocks if they self-reported multiple experiences. Questions were similar in each block but differed slightly between patients and HCPs. They are available via this link: https://www.york.ac.uk/media/healthsciences/documents/research/mentalhealthresearch/STIMULATE-ICP%20Delphi%20Survey%20Questions.pdf.

The research team initially organized responses (n = 283; responses = 1447) into four groups based on who made the statements: patients with experience of Long COVID (19 statements), patients with experience of long term conditions (10 statements), HCP with experience of Long COVID (14 statements) and HCP with experience of long term conditions (12 statements). This was reviewed and revised by small groups of expert panel members to ensure similar responses were grouped together and suggest summary statements which reflected the survey responses. To ensure all perspectives were considered in this process, each group meeting involved patient and clinician expert panel members, was chaired by a moderator panel clinician and observed by a moderator panel PPI representative. Feedback was gathered from the panels during meetings that were audio-recorded, with notes taken by an observer. Consensus discussions took place, the outcomes were recorded, and minutes circulated.

#### Round 2: establishing consensus and ranking

As the first round aimed at amassing as many relevant statements as possible, consensus was not calculated in round one. We calculated consensus levels on the statements in round 2 and defined consensus as at least 50% agreement in the response category. In case of lack of consensus, further rounds for achieving consensus were planned.

Expert panel members reviewed summary statements developed from Survey 1 responses during the second full expert panel meeting to remove any overlaps. In all rounds, feedback was gathered from the expert panel during expert panel meetings that were audio-recorded, with notes taken by an observer. Consensus discussions took place, the outcomes were recorded, and minutes circulated. The final list of 15 summary statements were presented as statements relevant to ICP for Long COVID and long term conditions in the second anonymous online survey. Survey participants were asked to respond to questions about health care priorities for Long COVID and long term conditions separately in blocks of questions so that all questions about Long COVID were together and all questions about long term conditions were together. To minimise response bias, these question blocks about Long COVID and long term conditions were presented in a random order; the list of statements within questions were also presented in a random order. Survey 2 asked participants to rate their level of agreement with each of the statements about Long COVID and long term condition care using a 5-point Likert scale (1. Strongly Agree, 5. Strongly Disagree). After this, participants were asked to prioritise the statements in order of importance (1 = most important, 15 = least important).

Median values were calculated to aid interpretation of the response category selected by survey participants. Interquartile deviation (IQD) values^61^ were used to ascertain consensus agreement from Survey 2 data (n = 197). IQD represent the spread of responses, with smaller values indicating greater consensus. Initially IQDs were calculated using a 50% threshold by determining the distance between the 25^th^ and 75^th^ percentiles (> 50% of individuals responded using the same category). IQD ≤ 1 was considered to indicate consensus as this indicates that cases fall within one response category from each other. Following discussion with the moderator panel who considered a Top-10 priority list to be optimal, statements which achieved 80% consensus (> 80% of individuals responded using the same category; n = 10 items) were included in prioritization. All response options were considered relevant for inclusion in this study. This means that items which achieved consensus for negative response options (Strongly disagree/Disagree) would be incorporated into the study results in the same way as positive response options (Strongly agree/Agree) were included; however, in this case consensus for all items were around positive response categories. Respondents were asked to prioritize all 15 summary statements in order of importance, placing the most important statement against the value of 1 and the least important statement against the value of 15. The sum of prioritization responses was used to establish the order of statements; the item with the lowest sum value was considered to have the highest priority.

#### Round 3: developing policy recommendations

Ten statements achieved 80% consensus after Survey 2; these were the same for both Long COVID and long term conditions. Further surveys to establish consensus were therefore not necessary. Full consensus and prioritization results are shown in Table 3.

The results of Survey 2 were presented in IQD format and discussed with the moderator panel in relation to Long COVID initially, and then in relation to long term conditions. Following this, the team decided to consider the recommendations together due to the similarities in priorities and rank order for both groups. The research team and moderator panel then worked together to develop the key domains relevant to the ten identified priorities and the statements which underpinned them. During these discussions, draft policy recommendations from the ten statements which achieved consensus were developed. The process of understanding the Survey 2 findings were then discussed with the expert panel in a final expert panel meeting. Priorities for Long COVID and their ranking were presented in IQD format, and then for long term conditions before discussion about the similarities and differences between the priorities and rank order. The research team and moderator panel process of developing the key domains and the draft policy recommendations was then also discussed with expert panel members. The scope of recommendations, appropriate stakeholders to share these with and co-signatories who may be interested in supporting the recommendations were also included in this discussion. The final recommendations from this work are organized into the four key domains: Diagnosis, Research, Resources and Training. Dissemination strategies for these recommendations were discussed with expert panel members and moderator panel members. Feedback was gathered from the expert panel during this meeting that was audio-recorded, with notes taken by an observer. Consensus discussions took place, the outcomes were recorded, and minutes circulated. The discussion continued until consensus was reached.

In addition to the expert panel and moderator panel members, representatives of relevant long term condition charities attended the second half of the final expert panel meeting. These representatives contributed to discussions concerning approaches to dissemination.

Priorities for health care of Long COVID and of long term conditions were initially examined independently, and then compared. The research team and moderator panel considered the priorities and underlying statements for these two groups, identifying key themes across all areas in order to develop key domains across all priorities. Draft policy recommendations were developed for the identified key domains using the same approach, prior to consultation with the expert panel members.

### Survey participants

Snowball sampling was used to recruit participants for both surveys. Existing clinical, patient support and social media networks shared the information to generate diverse samples with a variety of clinical experiences relevant to ICPs. To ensure diversity of sex and ethnicity, purposive sampling through the research platform PROLIFIC was incorporated into Survey 2 recruitment^57^.

Survey 1 (n = 283) focused on patient or health care professional experience of Long COVID or long term conditions. Questions for patients and HCPs differed slightly as indicated in the pdf via this link https://www.york.ac.uk/media/healthsciences/documents/research/mentalhealthresearch/STIMULATE-ICP%20Delphi%20Survey%20Questions.pdf.

Initially 328 people consented to complete Survey 1, however 45 were excluded for the following reasons: incomplete responses to eligibility items (n = 11), residing outside of the UK (n = 20), out of scope experiences (n = 14). Data presented for Survey 1 therefore relate to priorities for integrated care for Long COVID and long term conditions from 283 respondents (1447 suggestions). These did not reflect current clinical care provision but what respondents considered to be the most important aspects of care for these patient groups. Responses for Long COVID and long term conditions were organized separately initially, but due to the high level of overlapping themes, a single list of 15 statements was included in Survey 2. Survey 2 participants were asked to prioritize and rank from this list for both Long COVID and long term condition care independently.

**Figure 1:**
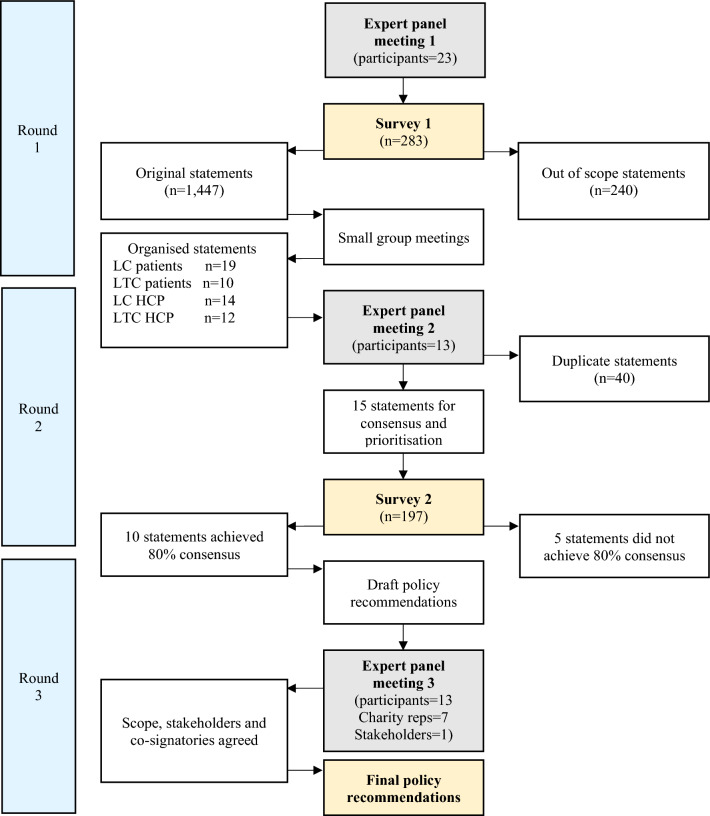
Flow diagram of policy recommendation development process. *LC* Long COVID, *LTC* Long term conditions, *HCP* Healthcare professional.

All UK-based adults were eligible to participate in Survey 2 (n = 197). Initially 485 people consented to complete Survey 2, however 288 were excluded for the following reasons: no response at all (n = 173), no demographic data to assess eligibility (n = 76), residing outside of the UK (n = 5), out of scope experiences (n = 34). Despite this, Survey 2 recruited a large sample (n = 197) diverse in terms of gender, age and ethnicity for consensus and prioritization of statements. Table 2 shows demographic characteristics for Surveys 1 and 2. The flow of participants through the study is shown in Fig. 1.

### Patient and public involvement (PPI)

Patients were involved in discussions and shaping of this work at all stages. PPI members were included in the expert panel and the moderator panel incorporating the experiences of people with a variety of Long COVID symptoms and long term conditions, and assisted with formulation of policy suggestions and dissemination of the results.

### Ethics

This research was conducted in accordance with the Declaration of Helsinki and was reviewed and approved by the University of York Health Sciences Research Governance Committee on 17·12·2021 (HSRGC/2021/478/A: STIMULATE). All survey participants provided informed consent.

### Preprint

A previous version of this manuscript was published as a preprint^62^

### Supplementary Information

Supplementary Information.

## Data Availability

Responses from Survey 1 data and the way these were organised and translated into Survey 2 statements are publicly available on website https://www.york.ac.uk/healthsciences/research/mental-health/projects/stimicp/*.* A minimal dataset from Survey 2 can be made available to interested parties upon request to the principal investigator. Researchers can submit a research plan, which describes the background and methods of a proposed research question, and a request for specific data of the database used for this study to answer the research question. Requests to access the datasets should be directed to christina.vanderfeltz-cornelis@york.ac.uk. The study protocol is available online^33^.
